# Vision: Its Optical Defects

**Published:** 1875-10

**Authors:** 


*Vision: Its Optical Defects and the Adaption of Spectacles.* By C. S. Fenner, M. D. Philadelphia: Lindsay & Blackiston, 1875.

The work under considration seems to have been written with a view to its adaptation to the general as well as to the medical reader. It will, therefore, not be expected to take as high a scientific standpoint as it would if intended solely for specialists in this department. The volume is divided into three parts: I. Physical Optics; II. Physiological; III. Errors of Refraction and Defects of Accommodation. An appendix gives instruction for the adaptation of spectacles. Selections from the Test Types of Snellen & Taeger are included in the work. The pages devoted to physical optics are an addition of great value to a work of this character, for an understanding of this department of physics is essential to a knowledge of what follows in the work. The eye as an optical instrument is well explained, and is a fair exposition of what we know concerning vision. The third section of the work is, perhaps, the most interesting to students of diseases of the eye, although the first two sections should by no means be neglected. In this section is included a description, illustrated by wood-cuts, of several of the most recent methods of determining the presence of astigmatism. Dr. Risley's method, by means of the *visnometer*, is probably the most recent of these. He places the far point at from ten to twelve inches, and hence it is necessary to have the accommodation thoroughly paralyzed in each case examined. Even then it would seem that errors might arise, owing to the proximity of the test to the eye. A full description of Dr. Risley's instrument, with illustrative cases, is given in the *American Journal of Medical Sciences* for October, 1875.

But little account is made in this section of errors of accommodation, almost the entire attention being given to errors of refraction

The author seems to be at home in the consideration of his subjects, and any of the few defects which may be noticed will readily be excused by the accident of retinal hemorrhage which befel him during the preparation of the work. The work seems in every regard worthy of the careful consideration of the profession.

